# Proteomic Identification and Quantification of Snake Venom Biomarkers in Venom and Plasma Extracellular Vesicles

**DOI:** 10.3390/toxins13090654

**Published:** 2021-09-15

**Authors:** Nicholas Kevin Willard, Emelyn Salazar, Fabiola Alejandra Oyervides, Cierra Siobhrie Wiebe, Jack Sutton Ocheltree, Mario Cortez, Ricardo Pedro Perez, Harry Markowitz, Anton Iliuk, Elda Eliza Sanchez, Montamas Suntravat, Jacob Anthony Galan

**Affiliations:** 1 National Natural Toxins Research Center (NNTRC), Texas A&M University-Kingsville, MSC 224, 975 West Avenue B, Kingsville, TX 78363, USA; nicholas.willard@students.tamuk.edu (N.K.W.); emelyn.salazarcastillo@tamuk.edu (E.S.); fabiola.oyervides@students.tamuk.edu (F.A.O.); cierra.wiebe@students.tamuk.edu (C.S.W.); jack.ocheltree@students.tamuk.edu (J.S.O.); mario.cortez@students.tamuk.edu (M.C.); elda.sanchez@tamuk.edu (E.E.S.); montamas.suntravat@tamuk.edu (M.S.); 2 Department of Chemistry, Texas A&M University-Kingsville, MSC 161, Kingsville, TX 78363, USA; 3 Omx Analytics, Kingsville, TX 38363, USA; omxanalytics@gmail.com; 4 Tymora Analytical Operations, West Lafayette, IN 47906, USA; rob@shannonscientific.com (H.M.); anton.iliuk@tymora-analytical.com (A.I.)

**Keywords:** snake venom biomarkers, extracellular vesicles, proteomics, EVtrap

## Abstract

The global exploration of snakebites requires the use of quantitative omics approaches to characterize snake venom as it enters into the systemic circulation. These omics approaches give insights into the venom proteome, but a further exploration is warranted to analyze the venom-reactome for the identification of snake venom biomarkers. The recent discovery of extracellular vesicles (EVs), and their critical cellular functions, has presented them as intriguing sources for biomarker discovery and disease diagnosis. Herein, we purified EV’s from the snake venom (svEVs) of *Crotalus atrox* and *C. oreganus helleri*, and from plasma of BALB/c mice injected with venom from each snake using EVtrap in conjunction with quantitative mass spectrometry for the proteomic identification and quantification of svEVs and plasma biomarkers. Snake venom EVs from *C. atrox* and *C. o. helleri* were highly enriched in 5′ nucleosidase, L-amino acid oxidase, and metalloproteinases. In mouse plasma EVs, a bioinformatic analysis for revealed upregulated responses involved with cytochrome P450, lipid metabolism, acute phase inflammation immune, and heat shock responses, while downregulated proteins were associated with mitochondrial electron transport, NADH, TCA, cortical cytoskeleton, reticulum stress, and oxidative reduction. Altogether, this analysis will provide direct evidence for svEVs composition and observation of the physiological changes of an envenomated organism.

## 1. Introduction

Snake venoms contain a diverse and extensive variety of toxins used to immobilize and digest their prey [1]. Though the diversity and composition of a snake’s venom can vary in toxicity and lethality from different species or within the same species, nearly all snake venom contains toxins from one of the twelve major proteins families [2]. These toxins are used to cause severe localized damage such as cell necrosis, hemolysis, edema, and inflammation that can later lead to hemorrhage, coagulopathy, and, without treatment, eventual death. Despite being considered the third most dangerous animal in the world (behind mosquitos and humans), and having a multitude of diverse toxic species spanning six continents, snake envenomings have been overlooked as a serious health concern [3]. Recently, the World Health Organization (WHO) has placed snake bite envenomation as a global health concern with 5.4 million snake bites causing 2.7 million cases, resulting on average 138,000 deaths a year and 400,000 cases of permanent disability [4]. Though the diversity of snakes and snake venom has been very well studied for many North and South American species, the detailed mechanism of action of snake envenomation remains poorly understood.

In the United States alone, it is estimated that there are about 10,000 snakebites per year that require emergency treatment [5]. About 4500 of these cases have been determined to be medically relevant and caused by snakes within the Crotalinae subfamily [5]. Several rattlesnakes from the Crotalinae are found within California, the most predominant being *C. oreganus* (Northern Pacific Rattlesnake). This species is found in the great central valley [6], and its subspecies *C. oreganus helleri* (Southern Pacific Rattlesnake), can be found in Southern California, Northern Baja California, and Mexico [7]. Another species of rattlesnake is the *Crotalus atrox* (Western Diamondback Rattlesnake), which accounts for most envenomations within northern Mexico and the United States [8]. Both *C. atrox and C. o. helleri* venoms are predominantly hemotoxic, myotoxic, cytotoxic, and hemorrhagic. Upon envenomation, the victim will suffer from severe pain, vomiting, edema, [9,10] and fluctuation of blood pressure [11]. The principal protein families found in *C. atrox* and *C. o. helleri* venom are L-amino acid oxidases (LAAOs), snake venom serine proteases (svSPs), snake venom phospholipase A_2_s (svPLA_2_s), and snake venom metalloproteinases (svMPs) [12,13]. Other proteins are also found, including cysteine-rich secretory proteins (CRiSPs), C-type lectins, and disintegrins. Indeed, the most abundant protein family is the svMPs, which can comprise of up to 70% of the total amount of protein in the venom [13].

These proteins found in snake venom are produced from a highly specialized gland that synthesizes, stores, and secretes the complex mixture of toxins. Many of these toxins are expressed as pro-enzymes in the active form or are kept inactivated by peptides liberated by prodomain hydrolysis or by other inhibitory factors present in the venom as the acidic pH environment, high citrate concentrations, and tripeptides containing pyroglutamate [14]. As such, envenomings result in highly active proteases which cleave basement membranes and non-enzymatic receptor antagonists (such as disintegrins and C-type lectins) that disrupt cell–cell interactions [14]. Despite venom originating from cells, very little attention has been given to the functionality of snake venom gland-derived extracellular vesicles (svEVs). Many cell types have the capability to release small membranous vesicles, including apoptotic bodies, microvesicles, and exosomes. Microvesicles can range from 150 to 500 nm and are formed by the outward budding and fission of the plasma membrane. Exosomes can range from 30 to 150 nm and are formed intracellularly by the inward budding of endocytic compartment membranes [15,16]. These EVs play a major role in many biological responses, such as cell communication, apoptosis, and immune-responses [17]. Recently, they have been given important attention due to the growing ability to be isolated from blood, urine, saliva, and breast milk using various analytical methods [18], and for their relevance in the quantification and identification of biomarkers in cancer, neurogenerative disease, cardiovascular disease, and infection [19,20,21,22,23].

Though svEVs were first observed in 1973 [24], only four recent studies have shown evidence for snake venom extracellular vesicles and partial characterization [25,26,27,28]; however, their precise protein content, function, and mechanism/role in snake envenomation remain unknown. In our study, we examined *C. atrox* and *C. o. helleri* snake venom-derived extracellular vesicles. Both displayed a unique venom toxin composition in EVs. Interestingly, EVtrap enrichment revealed previously unidentified signaling, adaptor, transmembrane, and vesicle proteins. To further explore EVs in *C. atrox* and *C. o. helleri* envenomation, EVtrap [29,30] and quantitative mass spectrometry were used to analyze mouse plasma-derived extracellular vesicles after sublethal injection. Our results shed new insights into snake venom extracellular vesicles and quantify potential biomarkers for snake envenomation resulting in altered metabolic pathways.

## 2. Results and Discussion

**Figure 1:**
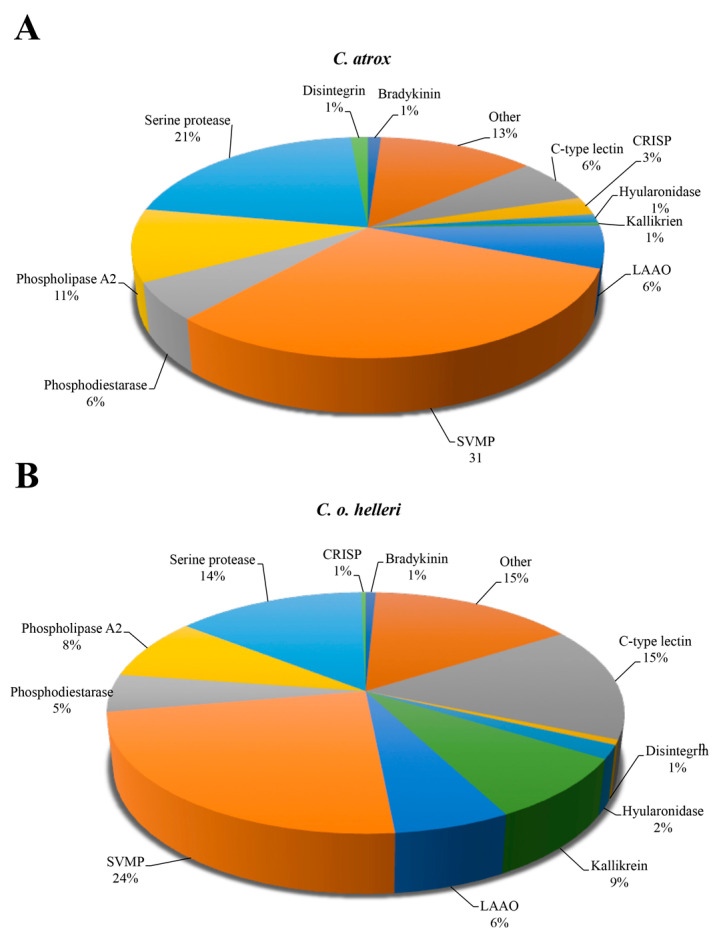
Proteomic analysis of the relative abundance of venom proteins in (**A**) *C. atrox* and (**B**) *C. o. helleri* venoms.

This study explored the proteomic identification and quantification of snake venoms and their biomarkers in extracellular vesicles utilizing mass spectrometry and quantitative proteomic approaches for the detection of svEVs and global systemic signature of snake envenomation. *C. atrox* and *C. o. helleri* were designated as medically important snakes contributing to the most bites and envenomations resulting in skin/tissue damage, muscle necrosis, perturbations in hemostasis, and possible limb loss due to the presence of highly abundant LAAO, svMPs, svSPs, and PLA_2_s [12,31] (Figure 1, Supplemental Tables S1A and S1B). The relative abundance of venom proteins was first established for both *C. atrox* and *C. o. helleri* by LC–MS/MS analysis. When compared, both venom proteomes had the following components: svMPs (*C. atrox* 31%, *C. o. helleri* 24%), svSPs (*C. atrox* 21%, *C. o. helleri* 14%), svPLA_2_s (*C. atrox* 11%, *C. o. helleri* 8%), C-type lectins (*C*. *atrox* 6%, *C. o. helleri* 15%), LAAO (*C. atrox* 6%, *C. o. helleri* 6%), and disintegrins (*C. atrox* 1%, *C. o. helleri* 1%) (Figure 1). As expected, and typical of *Crotalus* species, the most abundant protein family found in both *C. atrox* and *C. o. helleri* crude venoms were svMP, which primarily degrade structural extracellular matrix substrates such as collagen and fibrinogen and svSP, which are responsible for anticoagulant effects [2]. Both of these major venom constituents promote tissue damage and hemorrhaging [30].

**Figure 2:**
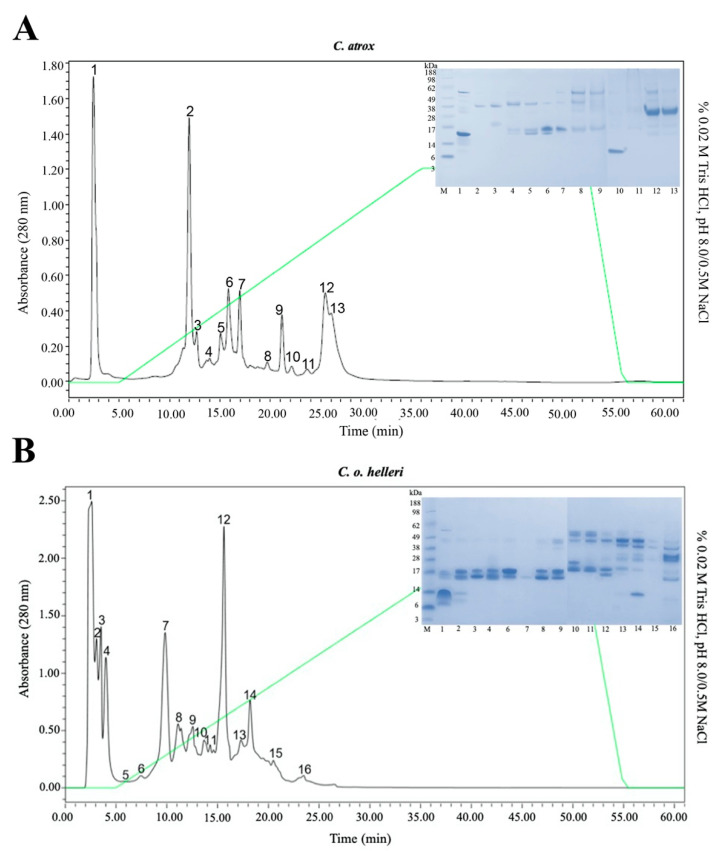
SDS-PAGE analysis of venom from Anion Exchange DEAE chromatography. A total of 5 μg of samples were run on a 4–12% Bis-Tris (MES) Gel (Novex^®^) at 100 V for 95 min. (**A**) *C. atrox*: Lane 1: SeeBlue^®^ Plus 2 prestained standard (1×); Lane 2: F1; Lane 3: F2; Lane 4: F3; Lane 5: F4; Lane 6: F5; Lane 7: F6; Lane 8: F7; Lane 9: F8; Lane 10: F9; Lane 11: F10; Lane 12: F13; Lane 13: F14. (**B**) *C. o. helleri*: Lane 1: SeeBlue^®^ Plus 2 pre-stained standard (1×); Lane 2: F1; Lane 3: F2; Lane 4: F3; Lane 5: F4; Lane 6: F6; Lane 7: F7; Lane 8: F8; Lane 9: F9; Lane 10: F10; Lane 11: F11; Lane 12: F12; Lane 13: F13; Lane 14: F14; Lane 15: F15; Lane 16: F16.

To further highlight the complexity of the *C. atrox* and *C. o. helleri* anion exchange, DEAE chromatography was utilized to fractionate crude *C. atrox* and *C. o. helleri* venoms. Electrophoretic profiles of these crude venom fractions were then developed via non-reduced SDS-PAGE for further characterization (Figure 2). The analysis revealed less complexity within the *C. atrox* venom than *C. o. helleri* when the two profiles were compared. The most prominent bands for the *C. atrox* crude venom gel fell in the range between 15 and 30 kDa, while those for *C. o. helleri* were observed from a broader range between 6 and 50 kDa (Figure 2). The LD_50_ for the *C. atrox* and *C. o. helleri* in our study was 5 mg/kg (0.95 mg/mL) and 3.3 mg/kg (0.62 mg/mL), respectively.

**Figure 3:**
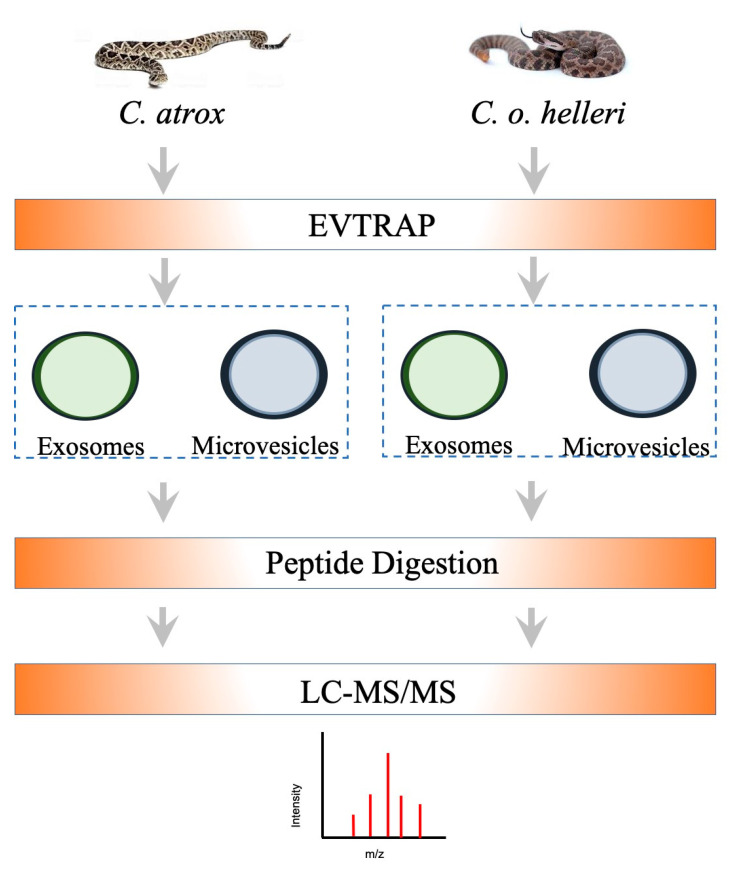
The proteomics workflow for svEVs isolation and analysis of venom from *C. atrox* and *C. o. helleri*. EVs, including microvesicles and exosomes, were isolated using EVtrap, followed by protein extraction, digestion, and enrichment for LC–MS analyses.

**Figure 4:**
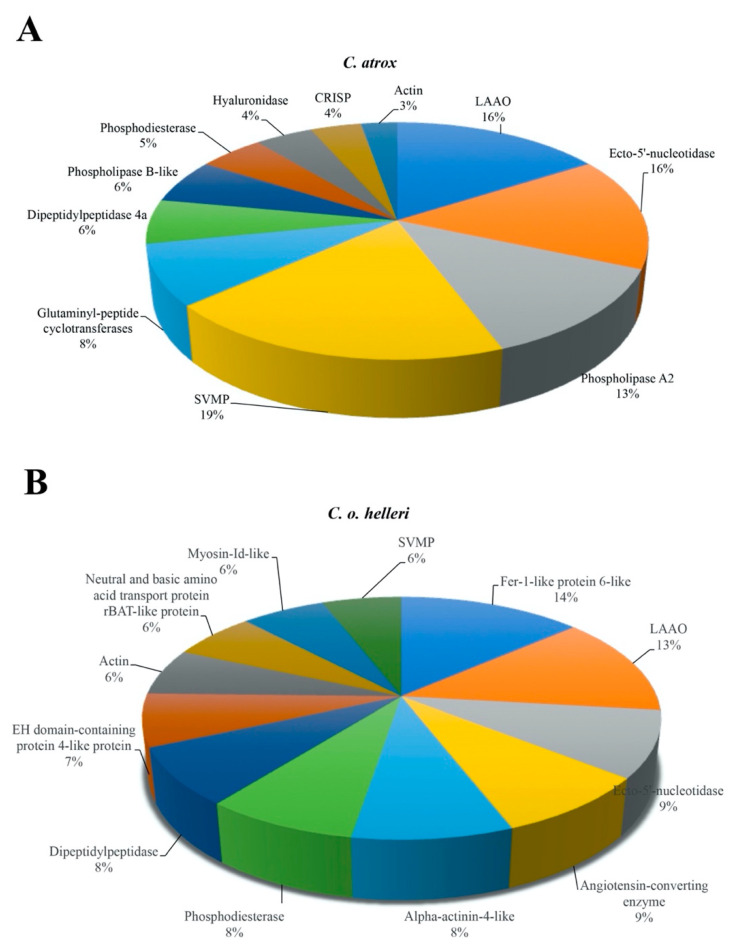
The proteomic analysis and the relative abundance of svEVs isolated from (**A**) *C. atrox* and (**B**) *C. o. helleri* venoms.

To address the identification and composition of svEVs using EVtrap and proteomics, the isolation and analysis of snake crude venoms from *C. atrox and C. o. helleri* was performed following the proteomic workflow outlined in Figure 3; then, further analyzed using high-resolution LC–MS/MS (Figure 4). In our analysis of svEVs (Figure 4 and Supplemental Table S2A,B), there was a significant enrichment in LAAO (*C. atrox* 17%, *C. o. helleri* 13%) and ecto-5′-nucleotidase (*C. atrox* 12%, *C. o. helleri* 9%). These results were similar to the work by Souza-Imberg et al. (2017), where the use of size exclusion chromatography found the enrichment of LAAO and ecto-5′-nucleotidase (9–12%) [28]. In a snake bite, LAAOs have an apoptotic function and, through complementary mechanisms with the serine proteases, disrupt the envenomated organism’s ability to maintain hemostasis [32,33], suggesting a possible function within the svEVs. In addition, as an oxidoreductase, these enzymes function as hemorrhagic and hemostasis-impairing toxins. It has been reported in vipers, rattlesnakes, and elapids that ecto-5′-nucleotidase aids in immobilizing and killing prey [34], as well as having anticoagulant activities [35]. SVMP- VAP2B was also identified and is well described in *C. atrox* venom. This family of toxic proteins function by impairing cell adhesion, hemostasis, the and inhibition of platelet aggregation [36,37].

Additionally, present in svEVs was phosphodiesterase, which has been well established in snake venom and whose functions include the induction of hypotension, inhibition of platelet aggregation, edema and paralysis [38]. The dipeptidylpeptidase family of enzymes was also found and has been previously reported in *Crotalus* venoms [39] as contributing to the activity of snake venom proteases and inactivating immune-modulating repair mechanisms. Interestingly, in svEVs, we found an angiotensin-converting enzyme, which is well-known to cleave bradykinin and promote inflammation [40]. Indeed, the svEVs contained a vesicle and transmembrane proteins which promote endocytosis to membranes in other cells [19,41] and may indirectly contribute to svEV toxicity. For example, in our analysis of svEVs, we found Myosin-Id-like and EH Domain-Containing Protein 4-like protein with a calcium-binding domain, both of which function in membrane mobility and may have an impact on cell communication [42]. We identified Fer-1-like Protein 4, which can have apoptotic characteristics in cancer, suppress epithelial-mesenchymal transition, e-cadherin, vimentin, and fibronectin; all of which participate in cell adhesion, communication, growth, and migration [43]. It is not unreasonable to speculate that svEVs contribute to toxic perturbations of major signaling molecules and pathways. For example, in pit vipers, adenosine can be released by dipeptidyl peptidase, ecto-5′-nucleotidase phosphodiesterase, and can suppress cardiac function [39].

Interestingly, svEVs may have evolved as a mechanism for long-term toxicity to aid in digestion, which can last months, but when humans are envenomated, this presents serious long-term complications such as pain, swelling, chronic kidney disease, and neurological effects [44]. Moreover, the svEVs could be unique to the family and species venom gland they originate from, presenting a diverse set of functions and signaling modes after envenomation. More studies are needed to explore the snake venom proteome and svEVs in parallel. These data shed light on a possible novel mode of snake envenomation. One can postulate that the venom toxicity and lethality aids in prey immobilization and digestion, and svEVs may also be facilitating these processes. These data demonstrate that protein families within the crude venom and snake venom extracellular vesicles differ and could have different effects on an envenomated organism. Moreover, these data are encouraging for further studies on svEVs in order to fully understand their function and role in snake envenomation. The venom toxins and contributing svEV components not only present an interesting hypothesis for toxicity and lethality, but also long-term effects seen in snake envenomation patients.

**Figure 5:**
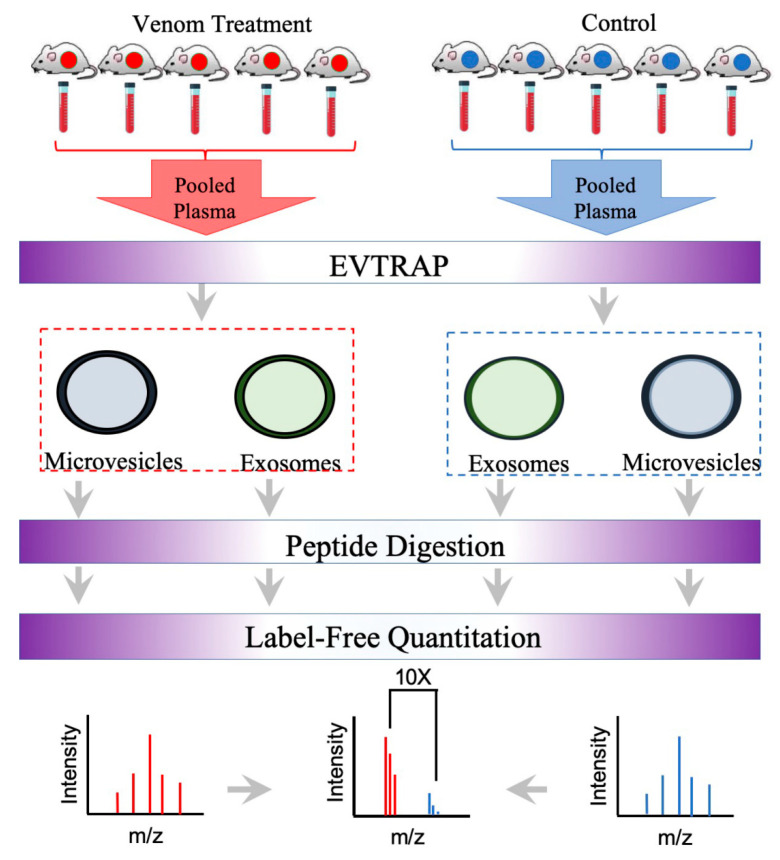
The proteomics workflow for plasma Evs from mice injected with venom from *C. o. helleri* and *C. atrox*. Evs were isolated using Evtrap, followed by protein extraction, digestion, and enrichment for LC–MS analyses.

Currently, the identification, composition, and role of circulating extracellular vesicles in snake bite envenomation victims remain unknown. Circulating extracellular vesicles have been reported in hemostatic disorders and pathophysiological thrombosis from the analysis of several cell-derived extracellular vesicles found in systemic circulation (such as red blood cells, platelets, leukocytes, and endothelial cells) [17]. Snake bite envenomation often results in mild to severe coagulopathy and alterations of physiological hemostasis and thrombosis [45,46], raising the possibility of circulating extracellular vesicles being present in victims. In order to address this question, as well as exploit the cellular features and capability of extracellular vesicles to harbor acute or chronic biomarkers of disease, EVtrap was utilized for the identification and quantification of biomarkers from EVs in plasma samples taken after snake bite envenomation. Exosomes derived from BALB/c mice treated with a sublethal dose of *C. atrox* and *C. o. helleri* crude venoms and purified via EVtrap were analyzed in a discovery-based initial screen to explore the venom-reactome following the proteomic workflow depicted in Figure 5.

**Figure 6:**
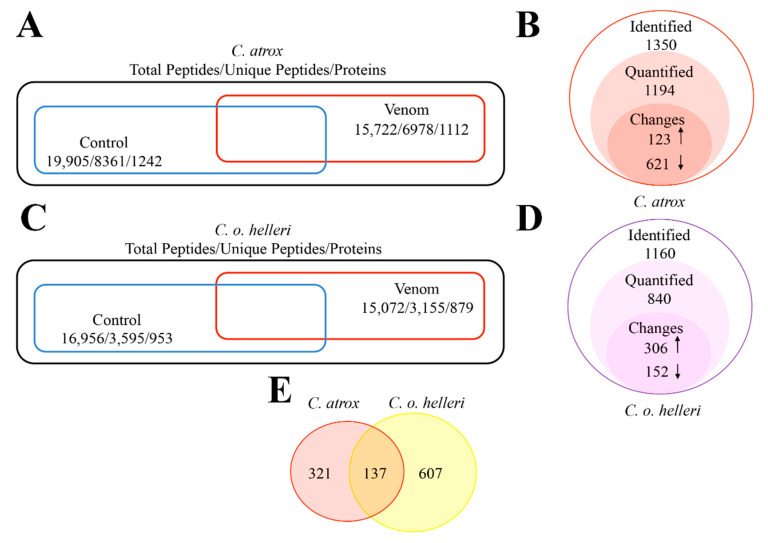
Schematic representation of the proteomic data form all experimental conditions. (**A**) Total proteins and peptides from *C. atrox* proteomic dataset. (**B**) Changes identified from label-free quantification in *C. atrox* dataset. (**C**) Total proteins and peptides from *C. o. helleri* proteomic dataset. (**D**) Changes identified from label-free quantification in *C. o. helleri* dataset. (**E**) The overlap of protein found between both snake envenomation *C. atrox* and *C. o. helleri* datasets.

**Figure 7:**
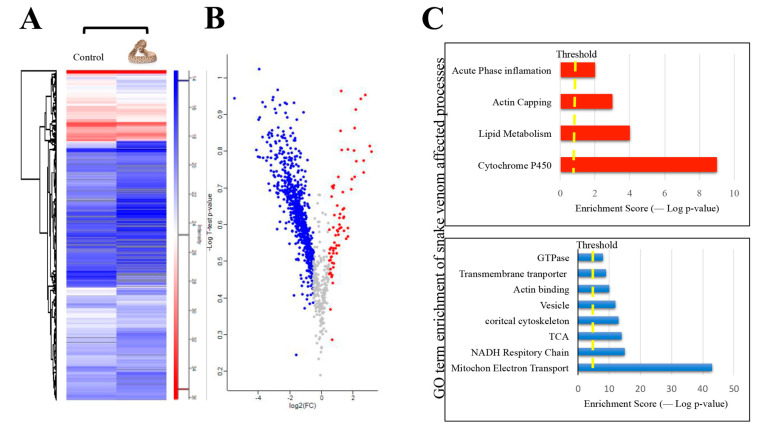
(**A**) The heat map of normalized abundances for differentially expressed proteins from plasma EVs between control sample of mice injected with PBS and mice injected with *C. atrox* venom. (**B**) Volcano plots showing the statistically differentially expressed proteins (*t*-test; FDR 0.05). The red represents a fold change greater than 0.1 and is considered upregulated, the blue represents a fold change of less than −0.5 and is considered downregulated, and the grey is unregulated proteins. (**C**) Gene ontology term enrichment of affected processes; the chart in red represents the most affected upregulated processes, and the bottom chart in blue represents the most affected downregulated processes.

An analysis of *C. atrox*-treated mouse plasma EVs revealed 1194 identifiable and quantifiable proteins. A total of 15,722 peptides were detected from EV-enriched mouse plasma. After label-free quantification, 1350 unique peptides with pairs (control and venom) were quantified, representing 1194 proteins (Figure 6A,B) (Supplemental Table S3A). The quantified results of these two experiments were volcano-plotted (Supplemental Table S4A) and a hierarchical cluster (Figure 7) using statistical methods. The resultant plots provided a depiction of the regulation of proteins based on a fold change. The analysis of *C. atrox*-treated groups found 123 upregulated and 621 downregulated proteins after venom treatment compared with the control (short list in Table 1 and Table 2; full list in Supplemental Table S5A).

The analysis of *C. o. helleri*-treated mouse plasma EVs revealed 840 identifiable and quantifiable proteins. A total of 15,072 peptides were detected from EV enriched mouse plasma. After label-free quantification, 1160 unique peptides with pairs (control and venom) were quantified, representing 840 proteins (Figure 6C,D). After removing proteins that were only represented in one group, there were 770 proteins remaining, which were, subsequently, used for a bioinformatics analysis (Supplemental Table S3B). There were 137 proteins commonly identified to both venom treatments (Figure 6E).

**Figure 8:**
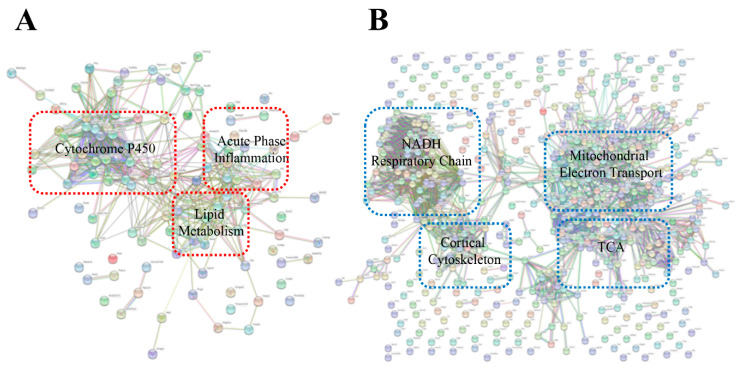
Analysis of protein–protein interactions (PPIs) against the STRING database for (**A**) upregulated proteins and (**B**) downregulated proteins from plasma EVs of mice injected with *C. atrox* venom.

The quantified results of *C.atrox*-treated proteomic data were mapped into a volcano plot (Supplemental Table S4A) and hierarchal clustering (Figure 7A–C). The resultant plots provided a depiction of the regulation of proteins based on a fold change. The DAVID and STRING bioinformatics software analysis showed that many of the upregulated response proteins were involved with cytochrome P450, lipid metabolism, and acute phase inflammation (Figure 8A), while downregulated proteins indicated an involvement with mitochondrial electron transport, NADH respiratory chain, the Tricarboxylic acid/citric acid cycle (TCA), and the cortical cytoskeleton (Figure 8B). It has been reported that snake venom can increase the formation of lipid droplets as part of inflammation mediation in snake envenomation [47]. Moreover, envenomation can result in a decrease of 60–70% in NADH and NADPH, suggesting snake venom proteins could directly affect mitochondrial levels and rates of the biosynthesis of NAD^+^ and NADP^+^, which may deplete the energy of the cell and, ultimately, lead to cell death [48].

**Figure 9:**
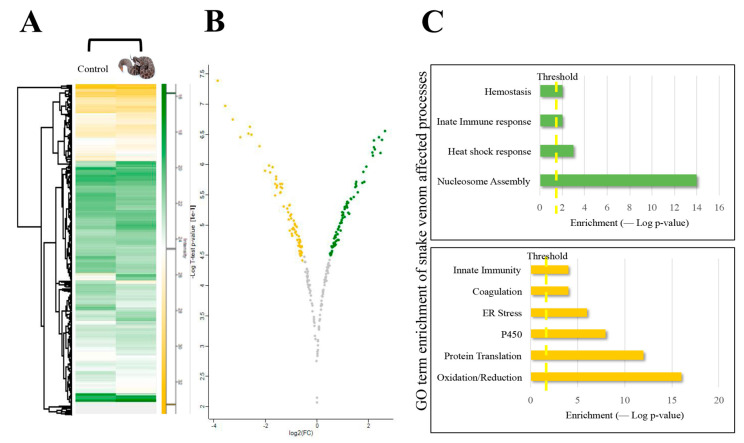
(**A**) The heat map of normalized abundances for differentially expressed proteins from plasma EVs between control sample of mice injected with PBS and mice injected with *C. o. helleri* venom. (**B**) Volcano plots showing the statistically differentially expressed proteins (*t*-test; FDR 0.05). The green represents a fold change greater than 0.1 and is considered upregulated, the yellow represents a fold change of less than −0.5 and is considered downregulated, and the grey is unregulated proteins. (**C**) Gene ontology term enrichment of affected processes; the chart in green represents the most affected upregulated processes, and the bottom chart in yellow represents the most affected downregulated processes.

The quantified results of *C.o. helleri* -treated proteomic data were mapped into a volcano plot (Supplemental Table S4B) and hierarchal clustering (Figure 9A–C). The resultant plots provided a depiction of the regulation of proteins based on a fold change. An analysis of *C. o. helleri*-treated groups found 306 upregulated and 152 downregulated proteins after venom treatment (short list in Table 3 and Table 4 full list in Supplemental Table S5B). Thus, one could postulate that the genetic composition was similar to a small extent, showing how close the snakes are related and, possibly, why the pathophysiological symptoms may seem similar. The snakes herein induce homeostatic disruption; however, the unique specificity details a novel mode of action. svEV’s may reduce or dampen the signaling to feedback mechanisms or reduce the energy; thus, limiting requirements for sustained cell survival. However, more studies are needed to test these observations.

**Figure 10:**
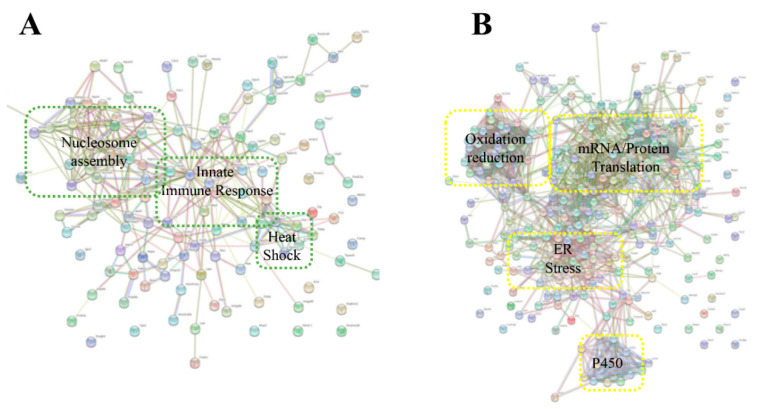
Analysis of protein–protein interactions (PPIs) against the STRING database for (**A**) upregulated proteins and (**B**) downregulated proteins plasma EVs of mice injected with *C. o. helleri* venom.

A bioinformatics software analysis showed that many of the upregulated responses were involved with cytochrome nucleosome assembly, an innate immune response, and heat shock (Figure 10A), while downregulated proteins indicated an involvement in oxidation reduction, protein translation, Endoplasmic Reticulum stress, and Cytochrome P450 (Figure 10B). *Crotalus* venoms have been well documented to be immunogenic [47], and have been shown to block the redox sites of myoglobin [49]. Further experiments are warranted to confirm these responses. These results may indicate unexplored and underlying cellular mechanisms of cell perturbation caused by svEVs in the pathophysiology of the snakebite. Indeed, snakebites from *Crotalus* species have similar clinical envenomation profiles such as in *C. atrox* and *C. o. helleri*. As expected, our data showed similarities in their EV content and systems level responses to envenomation. However, we identified potential modes of cell perturbation that may be unique to each snake.

## 3. Conclusions

A continued exploration into snake bite pathophysiology requires the use of multi-omics approaches to characterize the venom as well as the systemic response to venom as it enters the epidermis and blood stream of an organism. Venomics, the transcriptomics and proteomics of venoms, has led to recent advancements in the comprehension of snake venom composition both interspecies and intraspecies. As such, svEVs as a part of the sub-proteome of snake venom may be contributing to the envenomation process. More experiments are needed to test the function of svEVs. While these omics approaches give insights into the venom proteome, no method exists to analyze the reaction to venom after systemic circulation from the blood to explore the potential “venom-reactome”. Plasma is complex and may offer a rich source for analysis of potential snake venom biomarkers. A proteomic analysis of plasma cannot be easily carried out due to the large dynamic range, which spans 10^12^ of orders magnitude. However, disease biomarkers in endosomes and extracellular vesicles, which are purified from plasma, have shown exciting results. These technologies and analyses can be utilized to monitor the time course venom-reactome to snake envenomation, as well as the administration of antivenom after envenomation, giving a complete global signature of a snake bite and antivenom efficacy.

## 4. Materials and Methods

### 4.1. Venom Collection

Lyophilized Western Diamondback Rattlesnake (*C. atrox*) and Southern Pacific Rattlesnake (*C. o. helleri*) venom was obtained from the National Natural Toxins Research Center serpentarium located at Texas A&M University Kingsville, Kingsville, TX, and were designated as *C. atrox* vial 53 (AVID# 010-287-337) and *C. o. helleri* vial 792 (AVID# 046-536-058). Protein concentrations were determined by standard methods at 280 nm using an extinction coefficient of 1.

### 4.2. Snake Venom and Mouse Plasma Extracellular Vesicles Enrichment

svEVs were isolated using EVtrap [29,30]. Fifty milligrams of lyophilized venom were diluted in 1 mL of PBS and centrifuged at 10,000 rpm for 10 min to remove cellular debris. For non-lyophilized extracted venom, concentrated venom was diluted to 50 mg/mL, and 1 mL was centrifuged as stated above. The cleared venom was collected leaving the pellet behind. Samples were stored at −80 °C until ready to process. Magnetic EVtrap beads were provided by Tymora Analytical as a suspension in water. The EVtrap beads were added to the venom or plasma samples at 1:100 *v*/*v* ratio, and the samples incubated by shaking or end-over-end rotation for 1 h, according to manufacturer’s instructions. After supernatant removal using a magnetic separator rack, the beads were washed once with PBS and the EVs eluted by two 10 min incubations with 100 mM of fresh triethylamine (TEA, EMD Millipore, Burlington, MA, USA). The eluted samples were dried completely using a vacuum centrifuge.

### 4.3. Anion Exchange DEAE Chromatography 

Crude venom from *C. atrox* and *C. o. helleri* was fractionated by anion exchange DEAE chromatography. A total of 200 µL (8 mg) was fractioned using a WATERS™ Protein-Pak™ DEAE 5PW column (7.5 × 75 mm) (Milford, MA, USA). The column was equilibrated with 0.02 M Tris-HCl buffer, pH 8.0, and the fractions were eluted using 0.02 M Tris-HCl buffer containing 0.5 M NaCl, pH 8.0 over a period of 60 min with a flow rate of 1 mL/min. Eluted proteins were collected in 15 mL tubes, and a Breeze2 computer software system was used to generate the chromatogram. The absorbances of the fractions were read at 280 nm, and the tubes containing the fractions were stored at −20 °C until further use. 

### 4.4. Sodium Dodecyl-Sulfate Polyacrylamide Gel Electrophoresis (SDS-PAGE)

To identify the protein present in each fraction, all the fractions from all of the HPLC separation methods were ran using SDS-PAGE. Venom fractions were subjected to electrophoresis by NuPAGE^®^ Novex Bis-Tris gels (Invitrogen™, Carlsbad, CA, USA) under non-reducing conditions in an XCell SureLock Mini-Cell (Invitrogen Life Technologies, Waltham, MA, USA). A total of 5 µg of venom fractions were separated on a non-reduced NuPAGE^®^ Novex 4–12% (*w*/*v*) Bis-Tris gel for 95 min at a 100 V using an XCell SureLock^®^ Mini-Cell system (Invitrogen Life Technologies, USA). Current was moderated using a Bio-Rad PowerPack power supply. Gels were stained with 50 mL SimplyBlue SafeStain (Invitrogen Life Technologies, USA) for 24 h and distained overnight with 18 megaOhm water. SeeBlue Plus2 markers, ranging from 3 to 210 kDa, were used as standards. 

### 4.5. Subcutaneous Mouse Injection 

Male BALB/c mice (18–20 g) were subcutaneously injected in groups (*n* = 5) with 200 µL saline solution (control) or 0.95 mg/mL of *C. atrox* or 0.62 mg/mL of *C. o. helleri* crude venom (1 LD_50_). After 48 h, mice were sacrificed by cervical dislocation and blood was drawn by cardiac puncture and collected using 1% EDTA as anti-coagulant. Samples were processed within 60 min after blood extraction. Plasma was obtained by centrifugation at 4000 rpm for 10 min to remove platelets, debris, and large apoptotic bodies. The cleared plasma was collected leaving the pellet behind. Samples were stored at −80 °C until ready to process.

### 4.6. Ethical Procedures

All animal handling procedures were approved by the Texas A&M University Kingsville Institute of Animal Care and Use Committee (IACUC approval from hemorrhagic protocol (09-11-2018) #s 2018-11-09-A3).

### 4.7. Snake Venom and svEV Proteomic Analysis

*Snake venom:* one micrograms of snake venom proteins were denatured in 0.1% RapiGest (Waters, Milford, MA, USA) and reduced with 5 mM dithiothreitol for 30 min at 50 °C. Proteins were alkylated in 15 mM iodoacetamide for 1 h in the dark at room temperature and then digested with proteomics-grade trypsin at a 1:100 ratio overnight at 37 °C. The pH was adjusted below 3 and the sample was incubated for 45 min at 37 °C. The sample was centrifuged at 16,000× *g* to remove RapiGest. The supernatant was collected. The peptides were dissolved in 5 μL of 0.25% formic acid (FA) with 3% ACN. *svEV:* The isolated and dried EV samples were lysed to extract proteins using the phase-transfer surfactant (PTS)-aided procedure [50]. The proteins were reduced and alkylated by incubation in 10 mM trisphosphine (TCEP) and 40 mM chloroacetamide (CAA) for 10 min at 95 °C. The samples were diluted five-fold with 50 mM triethylammonium bicarbonate (TMAB) and digested with Lys-C (Wako, Richmond, VA, USA) at 1:100 (*wt*/*wt*) enzyme-to-protein ratio for 3 h at 37 °C. Trypsin was added to a final 1:50 (*wt*/*wt*) enzyme-to-protein ratio for overnight digestion at 37 °C. To remove the PTS surfactants from the samples, the samples were acidified with trifluoroacetic acid (TFA) to a final concentration of 1% TFA, and ethyl acetate solution was added at 1:1 ratio. The mixture was vortexed for 2 min and then centrifuged at 16,000× *g* for 2 min to obtain aqueous and organic phases. The organic phase (top layer) was removed, and the aqueous phase was collected. This step was repeated once more. The samples were dried in a vacuum centrifuge and desalted using Top-Tip C18 tips (GlyGen, Columbia, MD, USA) according to manufacturer’s instructions. 

### 4.8. LC–MS–MS

The samples were dried completely in a vacuum centrifuge and stored at −80 °C. One microgram of each dried peptide sample was dissolved in 10.5 μL of 0.05% trifluoroacetic acid with 3% (vol/vol) acetonitrile. In total, 10 μL of each sample was injected into an Ultimate 3000 nano UHPLC system (Thermo Fisher Scientific, Vantaa, Finland). Peptides were captured on a 2 cm Acclaim PepMap trap column and separated on a heated 50 cm column packed with ReproSil Saphir 1.8 μm C18 beads (Dr. Maisch GmbH, Ammerbuch, Germany). The mobile phase buffer consisted of 0.1% formic acid in ultrapure water (buffer A) with an eluting buffer of 0.1% formic acid in 80% (*vol*/*vol*) acetonitrile (buffer B) ran with a linear 60 min gradient of 6–30% buffer B at flow rate of 300 nL/min. The UHPLC was coupled online with a Q Exactive HF-X mass spectrometer (Thermo Fisher Scientific). The mass spectrometer was operated in the data-dependent mode, in which a full-scan MS (from *m*/*z* 375 to 1500 with the resolution of 60,000) was followed by MS/MS of the 15 most intense ions (30,000 resolution; normalized collision energy—28%; automatic gain control target (AGC)—2E4: maximum injection time—200 ms; 60 s exclusion).The raw files were searched directly against the *Crotalus* or *Mus musculus* available in UniProt with no redundant entries, using Byonic (Protein Metrics) and SEQUEST search engines loaded into Proteome Discoverer 2.3 software (Thermo Fisher Scientific). MS1 precursor mass tolerance was set at 10 ppm and MS2 tolerance was set at 20 ppm. Search criteria included a static carbamidomethylation of cysteines (+57.0214 Da) and variable modifications of oxidation (+15.9949 Da) on methionine residues and acetylation (+42.011 Da) at N-terminus of proteins. Search was performed with full trypsin/P digestion and allowed a maximum of two missed cleavages on the peptides analyzed from the sequence database. The false-discovery rates of proteins and peptides were set at 0.01. All protein and peptide identifications were grouped, and any redundant entries were removed. Only unique peptides and unique master proteins were reported.

### 4.9. Data Acquisition, Quantification, and Bioinformatics

All data were quantified using the label-free quantitation node of Precursor Ions Quantifier through the Proteome Discoverer v2.3 (Thermo Fisher Scientific, Vantaa, Finland). For the quantification of proteomic data, the intensities of peptides were extracted with initial precursor mass tolerance set at 10 ppm, minimum number of isotope peaks as 2, maximum ΔRT of isotope pattern multiplets—0.2 min—, PSM confidence FDR of 0.01, with hypothesis test of ANOVA, maximum RT shift of 5 min, pairwise ratio-based ratio calculation, and 100 as the maximum allowed fold change. The abundance levels of all peptides and proteins were normalized using the total peptide amount normalization node in the Proteome Discoverer. For calculations of fold change between the groups of proteins, total protein abundance values were added together and the ratios of these sums were used to compare proteins within different samples. To infer biological significance, all ratios showing a 1.5-fold change (ratio ≥ 1.5 or ratio ≤ 0.65) were required. Peptide distributions were analyzed with Excel. Perseus software (Version 1.6.2.1) was used to visualize the data from Excel. In the “Main” box, the abundance ratios, as well as the individual abundances of the venom and the control of the snake venoms, were inserted. In the “Text” box, protein accession and description were inserted. A log_2_ transformation was performed on the abundance ratio and individual abundances. All of the “NaN” values were removed from the abundance ratio. A minimum of three valid values in total were selected, and the heat map was generated. A one sample t-test was performed between the control and venom sample with a false discovery rate of 1%. The negative log t-test *p*-value and abundance ratio was used to create the volcano plot. Bioinformatics analysis was performed with DAVID and STRING Analysis tools described [51,52,53].

## Figures and Tables

**Table 1:** Up-regulation of potential biomarkers from mouse plasma after *C. atrox* envenomation.

Accession No.	Protein	Fold Change
Q05421	Cytochrome P450 2E1	>100
O88451	Retinol dehydrogenase 7	>100
Q4VAA2	Protein CDV3	>100
Q9CQW3	Vitamin K-dependent protein Z	>100
Q61316	Heat shock 70 kDa protein 4	>100
Q99KC8	von Willebrand factor A domain-containing protein 5A	>100
P50172	Corticosteroid 11-beta-dehydrogenase isozyme 1	>100
O88587	Catechol O-methyltransferase	>100
Q64152	Transcription factor BTF3	>100
A0A0R4IZY2	Cytochrome P450 2D26	>100
Q20BD0	Heterogeneous nuclear ribonucleoprotein A/B	>100
Q9R0H2	Endomucin	>100
P06880	Somatotropin	>100
P97429	Annexin A4	>100
P56654	Cytochrome P450 2C37	>100
P70296	Phosphatidylethanolamine-binding protein 1	>100
P56656	Cytochrome P450 2C39	>100
Q8K0C5	Zymogen granule membrane protein 16	>100
H3BKH6	S-formylglutathione hydrolase	>100

**Table 2:** Down-regulation of potential biomarkers from mouse plasma after *C. atrox* envenomation.

Accession No.	Protein	Fold Change
Q9QYG0	Protein NDRG2	>100
B7ZW98	Ank1 protein	>100
Q9Z239	Phospholemman	>100
Q8BYH8	Chromodomain-helicase-DNA-binding protein 9	>100
P22437	Prostaglandin G/H synthase 1	>100
Q9CZE3	Ras-related protein Rab-32	>100
Q8BP47	Asparagine--tRNA ligase, cytoplasmic	>100
A0A0A6YVW3	Protein Ighv1-23 (Fragment)	>100
Q80XI4	Phosphatidylinositol 5-phosphate 4-kinase type-2 beta	>100
Q91YE6	Importin-9	>100
D3YZZ5	Protein Tmed7	>100
Q9CR86	Calcium-regulated heat stable protein 1	>100
O70435	Proteasome subunit alpha type-3	>100
P61953	Guanine nucleotide-binding protein G(I)/G(S)/G(O) subunit gamma-11	>100
P01748	Ig heavy chain V region 23	>100
Q9Z1P6	NADH dehydrogenase (ubiquinone) 1 alpha subcomplex subunit 7	>100
Q3TVI8	Pre-B-cell leukemia transcription factor-interacting protein 1	>100
P56393	Cytochrome c oxidase subunit 7B, mitochondrial	>100
D3Z2F8	Actin-related protein 2/3 complex subunit 3	>100
O08795	Glucosidase 2 subunit beta	>100
A6PWS5	Gelsolin (Fragment)	>100
P62334	26S protease regulatory subunit 10B	>100
P55821	Stathmin-2	>100

**Table 3:** Up-regulation of potential biomarkers from mouse plasma after *C. o. helleri* envenomation.

Accession No.	Protein	Fold Change
A8DUK4	Beta-globin	>100
A0A0R4J0I9	Low-density lipoprotein receptor-related protein 1	>100
P01644	Ig kappa chain V–V region	>100
G3UXX3	Sepiapterin reductase	>100
A0A1W2P7F1	Complement component 1, s subcomponent 2	>100
B2RT14	UDP-glucuronosyltransferase	>100
Q01279	Epidermal growth factor receptor	>100
Q8R0Y6	Cytosolic 10-formyltetrahydrofolate dehydrogenase	>100
P55258	Ras-related protein Rab-8A	>100
Q9D1D4	Transmembrane emp24 domain-containing protein 10	>100
Q9QXF8	Glycine N-methyltransferase	>100
Q3TNA1	Xylulose kinase	>100
Q91YI0	Argininosuccinate lyase	>100
Q9R257	Heme-binding protein 1	>100
D3YYS6	Monoglyceride lipase	>100
P47738	Aldehyde dehydrogenase, mitochondrial	>100
Q91X52	L-xylulose reductase	>100
A0A0G2JDE1	Immunoglobulin-heavy variable V8-12 (Fragment)	>100
A0A1L1SSA8	Transmembrane protein 205 (Fragment)	>100

**Table 4:** Down-regulation of potential biomarkers from mouse plasma after *C. o. helleri* envenomation.

Accession No.	Protein	Fold Change
Q64514	Tripeptidyl-peptidase 2	>100
A0A140LHR4	Serpin H1 (Fragment)	>100
Q1XH17	Tripartite motif-containing protein 72	>100
P11404	Fatty acid-binding protein, heart	>100
Q8K274	Ketosamine-3-kinase	>100
E9Q4M2	Hormone-sensitive lipase	>100
Q9JHK5	Pleckstrin	>100
H3BKL6	Melanoma inhibitory activity protein 2	>100
Q64314	Hematopoietic progenitor cell antigen CD34	>100
Q920L1	Acyl-CoA (8-3)-desaturase	>100
Q9CVB6	Actin-related protein 2/3 complex subunit 2	>100
P08207	Protein S100-A10	>100
Q3B7Z2	Oxysterol-binding protein 1	>100
Q99MA9	Homeobox protein Nkx-6.1	>100
Q7TMY4	THO complex subunit 7 homolog	>100
F7BJH9	Predicted gene 21970 (Fragment)	>100

## Data Availability

Not applicable.

## Supplementary Materials

The following are available online at https://www.mdpi.com/article/10.3390/toxins13090654/s1, Supplemental Data S1: venom composition, Supplemental Data S2: venom EV composition, Supplemental Data S3: heatmap, Supplemental Data S4: plasma EV quantification.
